# Do fluorescent agents alter the mechanical strength of orthodontic adhesives? An *in vitro* and clinical study

**DOI:** 10.1186/s40510-020-0304-y

**Published:** 2020-02-10

**Authors:** Paulo Henrique Rossato, Edmilson Nobumito Kaneshima, Fábio Domingues, Thais Maria Freire Fernandes, Sandrine Bittencourt Berger, Paula Vanessa Pedron Oltramari

**Affiliations:** 1 Department of Oral Health, IFPR, Federal Institute of Parana (IFPR), Londrina, Brazil; 2 Department of Orthodontics, UNOPAR (University of North Parana), Londrina, Brazil; 3 Department of Restorative Dentistry, UNOPAR (University of North Parana), Londrina, Brazil

**Keywords:** Adhesive removal, Debonding, Bracket bond, UV light, Fluorescence, Shear bond strength, Adhesive remnant index

## Abstract

**Background:**

Fluorescent agents are added to orthodontic adhesives with the aim of making them visible under ultraviolet (UV) light, which ensures the complete, safe removal of remnants after orthodontic treatment. However, it is necessary to evaluate if the mechanical strength of these materials is maintained. Therefore, this study evaluated whether the addition of fluorescent agents influences the shear bond strength and clinical performance of a UV light-sensitive adhesive system.

**Methods:**

This study consisted of two stages: (1) *In vitro* phase: 40 human teeth were selected, divided at random into 2 groups (*n* = 20), according to the adhesive system used: UV group—adhesive with fluorescent agent, and control group—conventional adhesive. A shear bond strength test was performed using a DL 2000 universal testing machine, at a speed of 0.5 mm/min. The accessories were removed and an evaluation of the Adhesive Remnant Index (ARI) was carried out. (2) Clinical phase: 8 patients were selected and had their appliances bonded using the split-mouth design (160 teeth) with the same tested adhesive systems (UV, *n* = 80; control, *n* = 80). The patients were monitored for bonding failure for a period of 24 months. Statistical analysis was performed using the Independent *t* test, chi-squared tests, and Mann-Whitney test, at a level of significance of 5% and confidence interval of 95%.

**Results:**

Regarding the in vitro phase, the shear bond strength test yielded similar results in the two groups (*p* > 0.05) and the ARI showed statistically significant differences between the groups with a score of 1 being the most frequent ARI for both groups (70%). In addition, there was no clinical difference in terms of bonding failure between the groups (*p* > 0.05).

**Conclusion:**

The addition of fluorescent elements does not alter the mechanical strength and performance of the orthodontic adhesive and represents a viable alternative for clinical application.

## Background

The process of removing adhesive remnants after the debonding of orthodontic accessories remains a challenge for the orthodontist [1], as all the proposed techniques produce different degrees of polishing and come with some form of abrasion, accompanied by a varying degree of enamel loss [1–6]. Moreover, concerned about the removal of excess adhesive, some orthodontists end up leaving remnants of material on the surface of the tooth, which promotes an accumulation of plaque and the consequent increased risk of caries [7].

Optical characteristics of natural teeth, such as fluorescence, are determined by the interaction of light with dentin, enamel, and dental pulp. This property enables the structure to absorb light energy from ultraviolet radiation and be visually differentiated, allowing contactless detection for various materials [8]. Rare earth oxides, such as europium, terbium, cerium and ytterbium, are well-known fluorescent materials widely used in many fields of dentistry to differentiate between dental materials and the tooth surface on visual inspection [8].

With the aim of finding more efficient solutions and making adhesives visible for complete and safe removal after orthodontic treatment, chemical elements in powder form have been added to adhesives to increase fluorescence, which permits the use of UV light for viewing the remnants after the debonding of the accessories [9, 10].

**Fig. 1:**
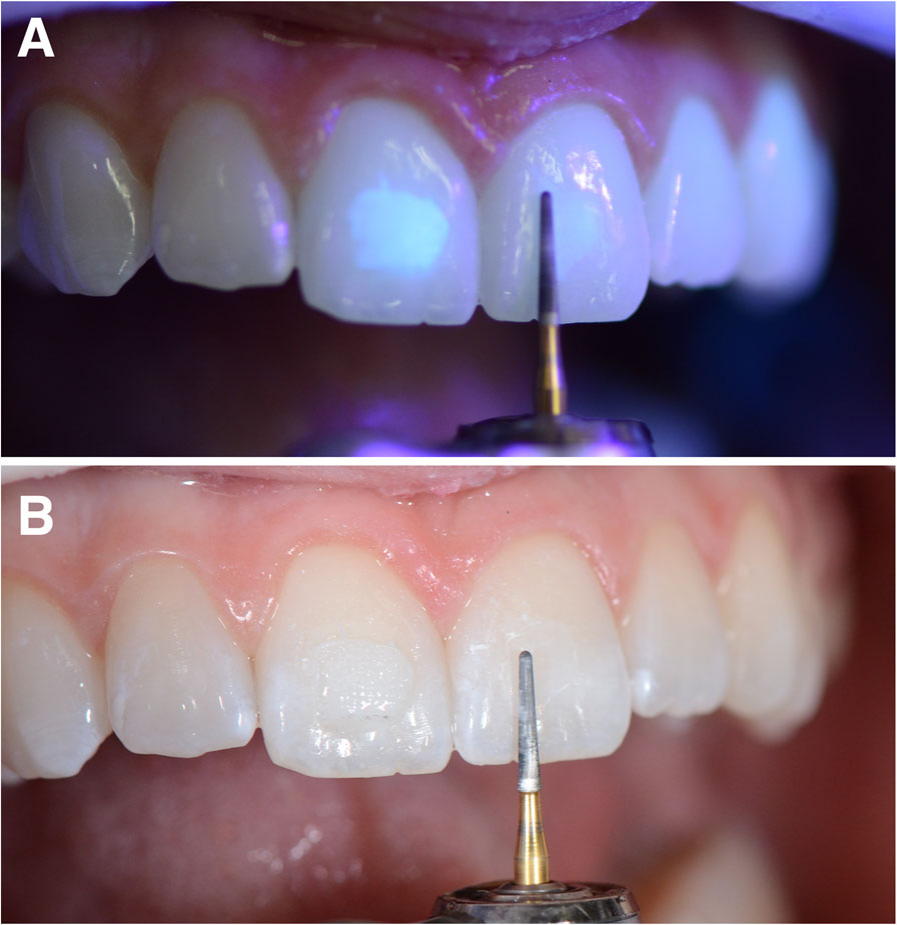
Clinical perspective of adhesive remnant after debonding with UV (**a**) and conventional (**b**) adhesives

From a clinical perspective, recent studies [10–13] have found that the use of UV light could help orthodontists view adhesives by using fluorescent agents, since they permit a more efficient removal of the remnants after debonding (Fig. 1a, b). This technology also reduces the length of time needed to carry out the procedure of removal of the adhesive remnants [12]. Another important clinical aspect is that adhesive systems for orthodontic use require a minimum load for shear bond strength [14].

As yet, no tests have been conducted to determine if the change in the composition of the adhesive, as a result of the incorporation of fluorescent agents, could have an impact on the material’s mechanical properties, such as shear bond strength, and have an adverse effect on the outcome of treatment. So, the aim of this study was to evaluate if the addition of fluorescent agents influences shear bond strength and clinical performance in a UV light-sensitive adhesive system during the debonding of orthodontic brackets.

## Material and methods

The Research Ethics Committee at the UNOPAR (University of North Parana) approved the protocol of this study.

This study comprised two stages: in vitro and clinical.

For the in vitro phase, a sample size of 20 premolar teeth was considered adequate for each group, based on a previous study [10]. Thus, a total of 40 human molars, referred for extraction, were selected. The teeth were cleansed and stored at approximately 5 ^°^C, immersed in deionized distilled water, thereby preventing any interference with the adhesion mechanism, for a maximum of one week prior to the tests. The inclusion criteria were as follows: absence of visible defects or damage caused by the extraction process or cracks in the enamel, absence of white spot lesions on the enamel, absence of caries and restorations on the vestibular surface of the enamel, absence of exposure to chemical products, and full root development [15, 16]. The root portions of the teeth were then embedded, one by one, in a PVC tube (diameter of 2 cm and height of 1.8 cm). The inside of the tubes was filled with self-curing acrylic resin so as to maintain the tooth in a central position, with the crown free, in order to permit the bonding of the accessories and position them with the vestibular surface in the perpendicular position. After the polymerization of the acrylic resin, the vestibular surfaces of all the samples were subjected to prophylaxis with a paste of pumice-stone and water, with the aid of a rubber cup, at low rotation for 10 s. The rubber cups were replaced every 5 teeth, which provided standardization in terms of the mechanical action of the prophylaxis for all the teeth. The teeth were then washed in running water for 10 s, and subsequently dried with light jets of oil-free, compressed air for 20 s [17]. The teeth received orthodontic tubes (3M Oral Care, Monrovia, CA, USA), bonded using the following adhesive systems: UV group (*n* = 20), Orthocem UV Trace adhesive with fluorescent agent (FGM, Joinville, Santa Catarina, Brazil); C group (*n* = 20), conventional adhesive (Transbond XT, 3M Oral Care). The accessories were cemented according to manufacturers’ instructions, as described in Table 1.

**Table 1:** Composition of the resins investigated

Material	Manufacturer	Composition: (% weight)	Application
Orthocem UV (UV group)	Dentscare Ltda. (Joinville, Brazil)	-Bisphenol A Diglycidyl ether methacrylate (BisGMA): 25–35 -Triethylene glycoldimethacrylate (TEGDMA): 10–15 -Phosphate methacrylate monomer: > 2 -Silanized silicon dioxide: 45–60 -Camphorquinone: < 1 -Sodium fluoride: > 1 -Fluorescent pigment: < 0.01	-Etching with 37% phosphoric acid for 15 s; -Continuous jet of water for 20 s; - Oil- and moisture-free jets of air; -Composite resin applied to the base of orthodontic accessory; -Polymerization for 20 s (10 s mesial and 10 s distal).
Transbond XT (C group)	3M Oral Care (Monrovia, CA, USA)	-Organic matrix: Bis-GMA and TEGDMA -Inorganic part: silica silanized with 70 to 80% by volume -n-dimethyl benzocaine, hexafluorphosphate -Camphorquinone	-Etching with 37% phosphoric acid for 15 s; -Continuous jet of water for 20 s; -Oil- and moisture-free jets of air; -Application of Transbond XT Primer adhesive (3M, Monrovia, CA, USA) and photo-polymerized for 30 s; -Application of Transbond XT at the base of the orthodontic accessory, which was polymerized for 20 s (10 s mesial and 10 s distal).

All the adhesive systems were polymerized using an SDI Radii-Cal curing light, with a peak light intensity of 1200 mW/cm^2^ (Dental Limited, Bayswater, Victoria, Australia), observing the manufacturer’s instructions. A single operator bonded all orthodontic tubes with a quantity of composite resin sufficient to cover the entire base of the accessories. The bonding was carried out in order to enable a careful fitting of the tubes in the center of the clinical crowns of the teeth (final position), applying firm pressure, so that the accessories were seated perfectly at the enamel surface. The excess adhesive around the entire base of these tubes was then removed [15, 18]. After bonding, the samples were stored in distilled water and kept in a 37 °C oven for 24 h [19]. At the end of this period, the test specimens were fitted in a cylinder with jaw clamps regulating their position so that the pivot point of the machine would execute a movement parallel to the vestibular surface of the teeth. The shearing tool was placed at the tube/enamel interface. The shear bond strength test was performed using a universal testing machine (DL 2000 EMIC), set to a speed of 0.5 mm/min, until the accessories were removed. The test results were obtained in N (Newtons) and converted to pressure values in MPa (Megapascal), verified based on the measurement of the base of the bracket used. After shearing, the vestibular surfaces of the samples were evaluated by two examiners (neither of whom were involved in the bonding procedure), in a blind analysis, in order to evaluate the Adhesive Remnant Index (ARI) [20] using the following system of scoring: 0—no adhesive remaining, 1—less than half of adhesive remaining, 2—more than half of adhesive remaining, 3—all adhesive remaining on the tooth surface. The viewing and evaluation of the tooth surfaces were carried out with the aid of a BVM100 microscope (BEL Photonics, Italy), at a magnification of up to × 10. Examiner calibration was carried out by means of repeated sample evaluation. The result was obtained by means of the consensus of the two examiners.

For the clinical phase, 8 patients (mean age 23.1 ± 2.3; 6 males and 2 females) requiring fixed orthodontic appliances were randomly enrolled. The inclusion criteria included class I malocclusion, absence of visible defects, damage or fissures, white spot lesions, caries, and restorations on the surface of the buccal enamel. Exclusion criteria were as follows: missing permanent teeth, supernumerary teeth, and prior orthodontic treatment. The study was based on a double-blinded split-mouth design, and the two adhesives were randomly applied on each side. All patients were blinded to the allocation of treatment and received brackets (3M Oral Care) that were bonded on one side with Orthocem UV Trace (FGM) (UV group, *n* = 80) adhesive with fluorescent agent, and on the other side with Transbond XT (3M/Oral Care) (control group), *n* = 80). The same operator bonded all the brackets, applying the same bonding technique, following manufacturers’ instructions (Table 1). It was not possible to blind the operators to the bonding system used because the two systems employed different methods of application. Patients were monitored over a 24-month period using the same wire sequence. A blinded external examiner assessed bonding failures for the data analysis.

### Statistical analysis

The laboratory results were analyzed through the independent *t* test. Clinical comparisons of bonding failures were analyzed via the chi-squared test with Yates correction with the Mann-Whitney test being used for assessing the ARI. All tests were analyzed at a level of significance of 5% and confidence interval of 95%, using BioEstat software.

## Results

With regard to the shear bond strength test, both adhesives produced similar results (*p* > 0.05) (Table 2).

**Table 2:** Shear bond strength (MPa) for both groups: mean, standard deviation (SD) and independent *t* test (*P*)

Shear bond strength (MPa)				
UVgroup (n = 30)		Control group (n = 30)		*p*
Mean	SD	Mean	SD	
12.69	5.05	13.80	4.55	> 0.05

**Fig. 2:**
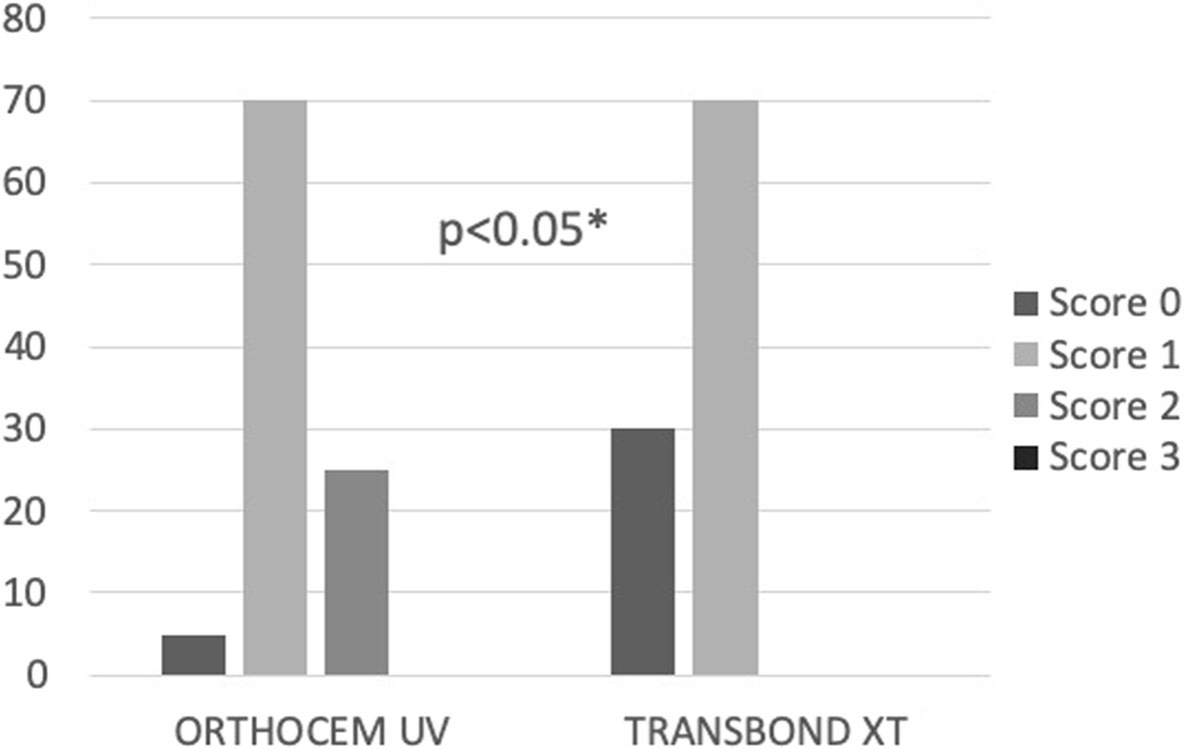
Graphic illustration showed statistically significant differences in the ARI scores between the groups (*p* = 0.022)

Figure 2 shows the observed frequency of ARI scores for each adhesive system tested. The test showed statistically significant differences in the ARI scores between the groups (*p* = 0.022). In the groups tested, no score 3 was observed and the highest frequency noted was for score 1 (70%).

Clinical results presented very low occurrences of failure (Table 3), with no significant difference between the groups. Bond failure occurred in 2.5% for the UV group and 5% for the control group, although this difference was not statistically significant (*p* > 0.05).

**Table 3:** Clinical performance (success and failure) in clinical bonding for UV group and control group: *n* and frequency (%) of occurrence, chi-squared test (*p*)

Group	Success *n*(%)	Failure *n*(%)	Total *n*(%)
UV	78 (97.5)	2 (2.5)	80 (100)
C	76 (95)	4 (5)	80 (100)
*p*	> 0.05		

## Discussion

It is known that all techniques employed for removing adhesive remnants (AR), following the debonding of brackets, produce different degrees of polishing and introduce some form of abrasion, accompanied by various degrees of enamel loss [1–6]. Thus, the quest for a safe, efficient method for the removal of AR, after the debonding of orthodontic accessories, has resulted in the introduction of a wide variety of instruments and procedures [1, 4, 21–23]. One possibility is the incorporation of fluorescent agents into adhesive systems capable of allowing the clinician to check the differences in fluorescence between the adhesive material and the enamel, thereby preserving the tooth surface [9, 13, 24, 25].

However, an important step in the verification of the efficiency of an adhesive system, for use in orthodontics, is the shear bond strength test [26, 27]. In the present study, this aspect was evaluated with particular attention due to the presence of an adhesive system with fluorescent agents (Orthocem UV trace, UV group), since the other one is a widely known system already used by professionals [28, 29]. The results demonstrated that the adhesive systems used did not show significant differences in terms of shear bond strength, with values close to the results previously described in the literature [28]. It should be emphasized that, despite the Orthocem UV Trace (UV group) being a two-step system, since it does not include the adhesive stage, this did not have an adverse effect on performance. Moreover, the presence of fluorescent agents in the UV group adhesive, similarly, had no impact on performance.

Rare earth oxides, widely used in dentistry to differentiate between dental materials and the tooth surface on visual inspection [8], have also been used to benefit orthodontic patients. The Eu^3+^ ion, one of the most popular lanthanides, is useful for the development of fluorescent, orthodontic adhesives as it emits monochromatic red light when excited with ultraviolet (UV) light. With the aim of looking for more effective solutions and making adhesives visible, for safe, complete removal after orthodontic treatment, Hamba et al. [9] added Eu^3+^ ions to yttrium oxide (Y^2^O^3^), which was a viable alternative for differentiation. Namura et al. [30], looked into the effect of incorporating different concentrations (0.001%, 0.002%, and 0.003%) of fluorescent dye derived from coumarin, in an adhesive system for bonding brackets to bovine teeth, and concluded that the adhesive containing 0.002% fluorescent dye possessed good shear bond strength (6.6 MPa), and also facilitated the removal of remaining material without impairing the structure of the enamel. The present study employed the UV system, though the manufacturer does not state what fluorescent agent is used, just the concentration (< 0.001% by mass). Therefore, this study obtained a shear bond strength greater than that of the abovementioned study [30] for all the adhesives tested, probably due to the bonding having been performed on human teeth.

The present study showed significant differences between groups for the ARI scores. Nevertheless, both groups exhibited higher frequencies of score 1 for the ARI (70%) in all systems tested, representing less than half of the adhesive remaining on the tooth surface after debonding, constituting a clinically desirable result because it reduced the amount of residual adhesive and the need for a rotary instrument for the cleanup [31]. However, a previous clinical study demonstrated that enamel damage regularly occurred during the debonding process with the extent of the damage being highly variable depending on the bracket material and adhesive system [32].

The laboratory results for both tested adhesives produced values above the recommended average, between 6 and 8 MPa, which represents the minimum load for the shear bond strength of an adhesive for clinical use in orthodontics [14]. It should also be stressed that highly accentuated shear bond strength could increase the risk of the enamel fracturing during the removal of the orthodontic accessories. Therefore, a suitable adhesive system for orthodontic use must be seen to be resistant to masticatory force [14] while preserving the dental structure in the accessory removal stage [32].

Additionally, the clinical results showed low failure rates for both adhesive systems tested (UV group = 2.5% and control group = 5%). Cal-Neto et al. [33] suggested that a failure rate below 10% is clinically acceptable. The UV adhesive system was compared to the gold standard in orthodontics and demonstrated similar clinical performance over a 24-month period. Despite the lack of evaluation at the end of the treatment, which is a limitation of the current study, the study was conducted considering that an average treatment time is 23.5 months [34].

Accordingly, this study compared a UV light-sensitive adhesive system to a conventional system, demonstrating no significant difference in shear bond strength and in clinical performance. These results show that the adhesive system with the fluorescent agent can be used and could deliver greater clinical practicality in the removal of adhesive remnants, particularly in posterior teeth, due to the differentiation of the optical characteristics of natural teeth. These characteristics could prevent potential damage to the surface layer of the enamel. Further investigation could be performed comparing the current adhesive with the fluorescent substance and the same material without it. This evaluation could address important information regarding the influence of fluorescent agents on the mechanical strength of orthodontic adhesives.

## Conclusion

- The addition of fluorescent elements does not alter the in vitro and clinical mechanical strength of the orthodontic adhesive;
- Adhesive systems with fluorescent agents represent a viable alternative for orthodontic use.

## Data Availability

All data generated or analyzed during this study are included in this published article. Please contact the corresponding author for requests for data.

## Abbreviations

AR: Adhesive remnant
ARI: Adhesive Remnant Index
CA: California
USA: United States of America
UV: Ultraviolet
